# Relationship Between Health Literacy and Unhealthy Lifestyle Behaviours in Older Adults Living in Switzerland: Does Social Connectedness Matter?

**DOI:** 10.3389/ijph.2023.1606210

**Published:** 2023-10-09

**Authors:** Maud Wieczorek, Clément Meier, Matthias Kliegel, Jürgen Maurer

**Affiliations:** ^1^ Swiss National Centre of Competence in Research LIVES—Overcoming Vulnerability: Life Course Perspectives, Lausanne and Geneva, Switzerland; ^2^ Faculty of Business and Economics (HEC), University of Lausanne, Lausanne, Switzerland; ^3^ Faculty of Biology and Medicine (FBM), University of Lausanne, Lausanne, Switzerland; ^4^ Swiss Centre of Expertise in the Social Sciences (FORS), University of Lausanne, Lausanne, Switzerland; ^5^ Centre for the Interdisciplinary Study of Gerontology and Vulnerability, University of Geneva, Geneva, Switzerland

**Keywords:** health literacy, lifestyle behaviours, social connectedness, older adults, SHARE

## Abstract

**Objectives:** To investigate the association between health literacy (HL) and unhealthy lifestyle behaviours and to explore the moderating role of social connectedness in this relationship in older adults in Switzerland.

**Methods:** We used data from 1,455 respondents to Wave 8 of the Survey of Health, Ageing, and Retirement in Europe (SHARE). Associations between the number of unhealthy lifestyle behaviours (smoking, risky alcohol consumption, suboptimal daily consumption of fruits/vegetables, lack of vigorous physical activity) and HL were examined using multivariable Poisson regression models, which allowed for interactions between HL and social connectedness to test the moderation hypothesis.

**Results:** Respondents with inadequate HL were significantly more likely to have a higher number of unhealthy lifestyle behaviours than respondents with sufficient HL. We found a stronger positive association between inadequate HL and the number of unhealthy lifestyle behaviours among socially isolated individuals.

**Conclusion:** Greater social connectedness seems to buffer the negative impact of inadequate HL on unhealthy lifestyle behaviours in older adults, highlighting the importance of good HL for healthy lifestyles, especially in individuals with low social reserve.

## Introduction

Lifestyle risk behaviours such as hazardous alcohol consumption, smoking, insufficient fruit and vegetable intake and lack of physical activity have been shown to account for a significant proportion of the global burden of disease and premature mortality [1, 2]. Recent literature suggested that a comprehensive strategy integrating nutrition and lifestyle changes would be the most effective way to increase the health span and, consequently, the quality of life of older adults [3]. Notably, the combination of four healthy lifestyle factors, namely, maintaining a healthy weight, exercising regularly, following a healthy diet, and not smoking, is associated with up to an 80% reduction in the risk of incident diabetes, myocardial infarction, stroke, or cancer in the general population [4]. As the beneficial effects of modifiable lifestyle behaviours, in particular physical activity and smoking avoidance, have been extended to healthy aging [5, 6], the adoption and adherence to healthy lifestyle behaviours remain of utmost importance in a context of an increasingly aging population [3].

Along with socioeconomic status, health and social service systems, physical and social environments, and cultural and personal determinants, health literacy has been recognised as a crucial concept in health promotion [7, 8]. Health literacy commonly refers to the knowledge, motivation, and competencies necessary to access, understand, appraise, and apply health information to make judgments and take decisions in everyday life concerning healthcare, disease prevention, and health promotion in order to maintain or improve quality of life during the life course [9]. Although some studies suggested relationships between health literacy and overall health status and mortality [10, 11], the exact mechanisms underlying these links and related heterogeneity across different population groups are not yet fully understood.

Lifestyle behaviours have been suggested as one of the pathways between health literacy and adverse health outcomes. So far, several cross-sectional studies have focused on the association between health literacy levels and modifiable lifestyle factors in the adult population but these studies often reported conflicting results [12–17]. Also, most of these studies focused on the respective roles of individual lifestyle factors, while evidence suggests that health behaviours are commonly co-occurring and tend to have cumulative beneficial or detrimental effects [18]. Last, to the best of our knowledge, few studies were conducted among older adults [19–22], and only one of them was conducted in the European older population [21], despite a particularly high risk of insufficient health literacy in older adults [23]. More insight into the associations between health literacy and lifestyle behaviours is thus needed to identify potential targets for interventions aimed at effectively mitigating the negative impact of insufficient health literacy in this vulnerable population group. Considering that individuals tend to draw on the health literacy of members of their social network [24] and the importance of the social environment for older adults’ overall health and wellbeing [25], social factors could be one of these targets to help buffer the adverse effects of insufficient health literacy [26, 27]. To the best of our knowledge, so far, only one study among older adults assessed the respective associations between health literacy and several health behaviours and whether different social factors moderate these associations [21]. However, these social factors reflect functional and quantitative characteristics of the social network rather than the qualitative aspects of the relationships maintained. Therefore, to fill the current knowledge gaps, the present study aimed to 1) investigate the relationship between health literacy levels and the number of unhealthy lifestyle behaviours in a population-based sample of older adults living in Switzerland and 2) explore the potential moderating role of both quantitative and qualitative aspects of social connectedness in this relationship.

## Methods

### Study Design and Participants

We used data from the Survey of Health, Ageing, and Retirement in Europe (SHARE), a multidisciplinary and longitudinal population-based survey of older adults aged 50 and older across 28 European countries and Israel [28]. At each biennial wave, data on health, socioeconomic status, social, family networks, and other life circumstances were collected using internationally harmonised computer-assisted personal interviews. In addition, participants were invited to complete a self-administered country-specific paper-and-pencil questionnaire.

The present study used data collected during the eighth wave of SHARE Switzerland, which took place between October 2019 and March 2020 [29, 30]. In total, 2,005 older adults living in Switzerland and their partners participated in the face-to-face interviews, and 94% of them (*n* = 1,891) also completed the Switzerland-specific paper-and-pencil questionnaire, which assessed respondents’ health literacy. At the time of sampling, SHARE Switzerland was designed to be nationally representative of community-dwelling individuals aged 50 and over. To maintain the sample’s representativeness, the last refreshment of the Swiss sample took place in 2011. As survey participants aged 50–58 in 2019/2020 could only enter SHARE as partners of target respondents, these survey participants were not representative of the general population aged 50–58. For this reason, the present study only included respondents, or their partners, aged 58 years and over in 2019/2020.

After excluding 114 respondents who did not complete the paper-and-pencil questionnaire, 28 respondents younger than 58 years old, and 294 respondents with one or more missing answers on the outcome, exposure variables, or covariates, the final analytical sample consisted of 1,455 individuals.

### Outcomes

Four unhealthy lifestyle behaviours were considered in the present study: current smoking, risky alcohol consumption, suboptimal daily consumption of fruits and vegetables, and lack of engagement in vigorous physical activity. Smoking was assessed by asking the respondents during the main interview if they were presently smoking cigarettes, pipe, cigars, cigarillos, or e-cigarettes with nicotine solution. Risky alcohol consumption was defined as the consumption of more than 12.5 units of alcohol per week [31]. Suboptimal daily consumption of fruits and vegetables was defined as consuming fruits and vegetables less than daily. Physical activity was assessed by asking the respondents how often they engaged in vigorous physical activity, such as sports, heavy housework, or a job that involves physical labour. The lack of engagement in vigorous physical activity was defined as being engaged in vigorous physical activity less than once a week. Each individual behaviour was coded into a binary variable (0 = absence, 1 = presence) to estimate the co-existence of unhealthy lifestyle behaviours. We then calculated the number of prevalent unhealthy lifestyle behaviours by adding up the binary variables for each respondent. This number ranged from 0 to 4, with 0 reflecting the absence of unhealthy behaviour and 4 representing the presence of all four unhealthy lifestyle behaviours.

### Exposures

The Switzerland-specific paper-and-pencil questionnaire assessed health literacy with the short version of the European Health Literacy Survey questionnaire (HLS-EU-Q16) [32]. This questionnaire consists of 16 items related to concrete health-relevant tasks or situations that respondents rate using a four-point Likert scale ranging from “very easy,” “fairly easy,” “fairly difficult,” to “very difficult.” As described by Pelikan et al., each item was dichotomized, with a value of “0” for the categories “fairly difficult” and “very difficult” and a value of “1” for “very easy” and “fairly easy” [33]. If the overall number of item non-response did not exceed two, missing item values were replaced by 0 [34]. The subjective health literacy total score was calculated by summing the values of each item only for respondents who answered at least 14 items and ranged from 0 to 16 [34]. Three categories of subjective health literacy levels were derived from the total score: inadequate health literacy levels (0–8), problematic health literacy levels (9–12), and sufficient health literacy levels (13–16) [33].

### Potential Moderator

Social connectedness was assessed with the social connectedness scale [35, 36]. This scale includes five main characteristics of the social network into a composite measure to capture the key facets of social network resources in a single indicator. Network size was determined by asking respondents the number of people that are important to them, proximity by the number of cited social network members living within 25 km, contact frequency by the number of cited people who have weekly or more frequent contact, and support the number of cited people who have very or extremely close emotional ties. Network diversity was determined by the number of different types of relationships (spouse, other family members including children, friends, and others) that were present in the network. Each item has a maximum of four points and the total raw score ranging from 0 to 20 was condensed into a calibrated measure between 0 and 4, with higher scores reflecting stronger network resources. Following an approach suggested by Beridze et al., we inverted the scale and defined social isolation with a score equal to or greater than 3 [37].

### Covariates

Additional covariates considered in the present study were socio-demographic variables, including sex (men, women) and age group (58–64 years, 65–74 years, 75+ years). Education levels were grouped into three categories based on the International Standard Classification of Education (ISCED) of 2017 (low, medium, high) [38]. The subjective financial situation of respondents was assessed based on the question: “Is your household able to make ends meet?”. Response categories were recoded as “easily,” “fairly easily,” and “with difficulty.” The variable related to respondents’ living area was dichotomised (urban, rural). The language used to answer the questionnaire (German, French, Italian) was used as a proxy for regional/cultural differences. We additionally considered three health characteristics as covariates: self-rated health (poor or fair health, good health, very good or excellent health), the prevalence of limitations in at least one activity of daily living (yes, no), and the presence of a major chronic disease including heart disease, diabetes, lung disease and cancer (yes, no).

### Statistical Analysis

The characteristics of the analytical sample were described using number counts and proportion estimation with corresponding 95% confidence intervals (CI). The distribution of unhealthy lifestyle behaviours by the three categories of health literacy levels was examined using mean and corresponding standard errors (SE). The Kruskal-Wallis test was used to assess the bivariate associations between health literacy levels and the number of unhealthy lifestyle behaviours. The partial associations between health literacy total score, health literacy levels, and the number of unhealthy lifestyles were examined separately using Poisson regression models. We performed chi-squared tests to evaluate the models’ fit with the data and finalized our model selection by comparing the residual plots from Poisson and negative binomial regression models to check overdispersion. The multivariable models, thereby, accounted for sex, age groups, education levels, subjective financial situation, living area, Swiss linguistic regions, self-rated health, limitations in activities of daily living, and the presence of major chronic conditions. Results were reported as average partial effects (APE) along with corresponding SE. For the continuous health literacy score, the APEs represent the average difference in the expected count of unhealthy lifestyle behaviours for every one-point increase in the health literacy score, keeping all other covariates constant. When examining the levels of health literacy, the APEs indicate the average difference in the expected count of unhealthy lifestyle behaviours when comparing inadequate or problematic levels to the reference level (sufficient), holding all other covariates constant.

We tested the moderation hypothesis by entering an interaction term between social connectedness and health literacy levels (total score and three categories) in the multivariable models including additionally social isolation as a main effect. We checked the statistical significance of the interaction term using the Wald test. A stratified analysis was conducted by category of social connectedness in case of significant interaction term. Since both respondents and their partners could be part of the SHARE study, the possibility of unobserved dependencies between two observations was accounted for in the multivariable models by clustering the estimated standard errors at the household level. Statistical analyses were conducted using STATA/SE 17.0 (STATA Corporation, College Station, TX, United States). Two-sided *p*-values < 0.05 were considered statistically significant.

## Results

### Main Characteristics of the Analytical Sample

Characteristics of the 1,455 respondents included in the analytical sample are described in Table 1. Most respondents were female (52.6%), and 40.9% were 65–74 years. The large majority had a medium education level (63.6%), could make ends meet easily or fairly easily (54.6% and 32.8%, respectively), lived in the German-speaking part of Switzerland (71.5%), and lived in a rural living area (54.6%). Regarding respondents’ health characteristics, 93.0% of the respondents did not have any limitations in activities of daily living, and almost half of them reported good health (41.6%).

**TABLE 1:** Main characteristics of the analytical sample, adults aged 58+, Survey of Health, Ageing, and Retirement in Europe, 2019/2020, *n* = 1,455.

		*n*	%	95% CI
Sex	Men	690	47.4	44.9, 50.0
	Women	765	52.6	50.0, 55.1
Age groups	58–64 years	359	24.7	22.5, 27.0
	65–74 years	595	40.9	38.4, 43.4
	75+ years	501	34.4	32.0, 36.9
Education levels	Low	247	17.0	15.1, 19.0
	Medium	926	63.6	61.1, 66.1
	High	282	19.4	17.4, 21.5
Make ends meet	Easily	794	54.6	52.0, 57.1
	Fairly easily	477	32.8	30.4, 35.2
	With difficulty	184	12.6	11.0, 14.5
Swiss linguistic regions	German	1,041	71.5	69.2, 73.8
	French	359	24.7	22.5, 27.0
	Italian	55	3.8	2.9, 4.9
Living area	Urban	660	45.4	42.8, 47.9
	Rural	795	54.6	52.1, 57.2
Self-rated health	Poor/Fair	285	19.6	17.6, 21.7
	Good	606	41.6	39.1, 44.2
	Very Good/Excellent	594	38.8	36.3, 41.3
Limitations in activities of daily living	Yes	102	7.0	5.8, 8.4
	No	1,353	93.0	91.6, 94.2
Prevalent major chronic disease(s)	Yes	313	21.5	19.5, 23.7
	No	1,142	78.5	76.3, 80.5
Health literacy levels	Sufficient	1,010	69.4	67.0, 71.7
	Problematic	329	22.6	20.5, 24.8
	Inadequate	116	8.0	6.7, 9.5
Social connectedness	Social isolation	300	20.6	18.6, 22.8
	No social isolation	1,155	79.4	77.2, 81.4

Abbreviation: CI, confidence intervals.

Overall, the mean number of prevalent unhealthy lifestyle behaviours was 1.2 (95% CI 1.1, 1.3). The numbers and proportions of respondents with one and two missing HLS-EU-Q16 items were 84 (5.8%) and 24 (1.6%), respectively. The respective prevalence of problematic and inadequate health literacy levels was 22.6% and 8.0%. One-fifth of our sample (20.6%) was considered to be socially isolated.

### Bivariate Associations Between Health Literacy and the Number of Unhealthy Lifestyle Behaviours

**FIGURE 1:**
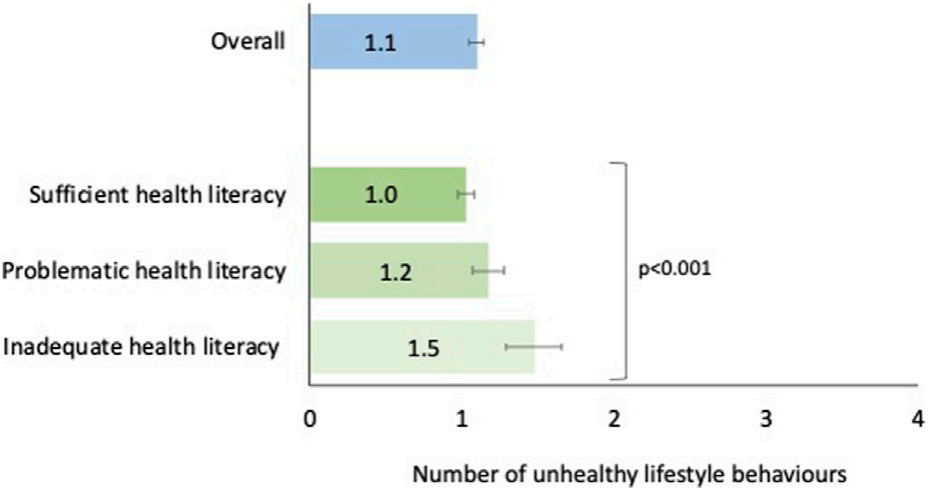
Mean number of unhealthy lifestyle behaviours with corresponding 95% confidence intervals—overall and by health literacy level, adults aged 58+, Survey of Health, Ageing, and Retirement in Europe—Switzerland, 2019/2020, *n* = 1,455.

Figure 1 shows the mean number of unhealthy lifestyle behaviours stratified by health literacy level. We found that respondents with problematic and inadequate health literacy levels had a significantly higher mean number of unhealthy lifestyle behaviours than their counterparts with sufficient health literacy levels (*p* < 0.001).

### Multivariable Associations Between Health Literacy and the Number of Unhealthy Lifestyle Behaviours

The partial associations between health literacy and the number of unhealthy lifestyle behaviours from adjusted multivariable models are presented in Table 2. When controlling for key sociodemographic and health-related variables, higher health literacy scores were significantly associated with having a lower number of unhealthy lifestyle behaviours (APE = −0.02, *p* < 0.01). Also, respondents with inadequate HL levels were significantly more likely to have a higher number of unhealthy lifestyle behaviours than respondents with sufficient health literacy levels (APE = 0.21, *p* < 0.05), holding other characteristics fixed.

**TABLE 2:** Partial associations between health literacy and the number of unhealthy lifestyle behaviours, adults aged 58+, Survey of Health, Ageing, and Retirement in Europe, 2019/2020, *n* = 1,455.

	Number of unhealthy lifestyle behaviours	Number of unhealthy lifestyle behaviours
	Model 1	Model 2
**Covariates**		
Sex	−0.03 (0.05)	−0.03 (0.05)
Women (vs. men)		
Age groups		
65–74 years (vs. 58–64)	−0.07 (0.06)	−0.07 (0.06)
75+ years (vs. 58–64)	0.03 (0.07)	0.03 (0.07)
Linguistic region		
French-speaking (vs. German-speaking)	−0.01 (0.05)	−0.01 (0.05)
Italian-speaking (vs. German-speaking)	−0.02 (0.11)	−0.02 (0.11)
Education levels		
Secondary (vs. low)	0.03 (0.06)	0.03 (0.06)
Tertiary (vs. low)	−0.01 (0.08)	−0.02 (0.08)
Make ends meet		
Fairly easily (vs. easily)	0.12* (0.05)	0.13* (0.05)
With difficulty (vs. easily)	0.21** (0.07)	0.22** (0.07)
Living environment		
Rural (vs. urban)	−0.04 (0.05)	−0.04 (0.05)
Self-rated health		
Good (vs. poor/fair)	−0.24*** (0.07)	−0.25*** (0.07)
Very good/excellent (vs. poor/fair)	−0.42*** (0.08)	−0.43*** (0.08)
Limitations in activities of daily living		
One or more (vs. no limitations)	0.24** (0.08)	0.24** (0.08)
Prevalent major chronic diseases		
Yes (vs. no)	0.04 (0.06)	0.05 (0.06)
**Exposure variables**		
Health literacy total score (0–16)	−0.02** (0.01)	
Health literacy levels		
Problematic (vs. sufficient health literacy)		0.05 (0.06)
Inadequate (vs. sufficient health literacy)		0.21* (0.09)

The table shows average partial effects and standard errors in parentheses from separate Poisson regression models for health literacy total score (Model 1) and health literacy levels (Model 2).

Statistical significance: *p < 0.05; **p < 0.01, ***p < 0.001.

Bold values indicate statistically significant *p*-values for the exposure variables.

### Moderating Role of Social Connectedness

Table 3 describes the partial associations between health literacy, social isolation and the number of unhealthy lifestyle behaviours from adjusted multivariable models. The interaction term between the health literacy total score and categories of social connectedness included in the multivariable model was statistically significant (*p* < 0.001), suggesting a significant moderating role of social connectedness in the association between health literacy total score and the number of unhealthy lifestyle behaviours. The results from the stratified analysis by category of social connectedness are shown in Table 4. Higher health literacy total scores were negatively associated with the number of unhealthy lifestyle behaviours in both categories of social connectedness, with a somewhat stronger association in respondents who were considered socially isolated (APE = −0.03, *p* < 0.05). Consistently, we found a significant interaction between categories of health literacy levels and categories of social connectedness (*p* = 0.04). Inadequate health literacy levels were significantly and positively associated with a higher number of unhealthy lifestyle behaviours, only among socially isolated individuals (APE = 0.34, *p* < 0.05).

**TABLE 3:** Partial associations between health literacy, social isolation and the number of unhealthy lifestyle behaviours, adults aged 58+, Survey of Health, Ageing, and Retirement in Europe, 2019/2020, *n* = 1,455.

	Number of unhealthy lifestyle behaviours	Number of unhealthy lifestyle behaviours
	Model 1	Model 2
**Covariates**		
Sex	−0.02 (0.05)	−0.02 (0.05)
Women (vs. men)		
Age groups		
65–74 years (vs. 58–64)	−0.07 (0.06)	−0.07 (0.06)
75+ years (vs. 58–64)	0.03 (0.07)	0.03 (0.07)
Linguistic region		
French-speaking (vs. German-speaking)	−0.00 (0.06)	−0.00 (0.06)
Italian-speaking (vs. German-speaking)	−0.06 (0.11)	−0.06 (0.11)
Education levels		
Secondary (vs. low)	0.03 (0.06)	0.03 (0.06)
Tertiary (vs. low)	−0.01 (0.08)	−0.02 (0.08)
Make ends meet		
Fairly easily (vs. easily)	0.12* (0.05)	0.12* (0.05)
With difficulty (vs. easily)	0.21** (0.07)	0.21 (0.07)
Living environment		
Rural (vs. urban)	−0.03 (0.05)	−0.04 (0.05)
Self-rated health		
Good (vs. poor/fair)	−0.24*** (0.07)	−0.25*** (0.07)
Very good/excellent (vs. poor/fair)	−0.41*** (0.08)	−0.43*** (0.08)
Limitations in activities of daily living		
One or more (vs. no limitations)	0.23** (0.08)	0.24** (0.08)
Prevalent major chronic diseases		
Yes (vs. no)	0.05 (0.06)	0.05 (0.06)
**Moderator**		
Social isolation	0.08 (0.06)	0.08 (0.06)
Yes (vs. No)		
**Exposure variables**		
Health literacy total score (0–16)	−0.02** (0.01)	
Health literacy levels		
Problematic (vs. sufficient health literacy)		0.05 (0.06)
Inadequate (vs. sufficient health literacy)		0.19* (0.09)

The table shows average partial effects and standard errors in parentheses from separate Poisson regression models for health literacy total score (Model 1) and health literacy levels (Model 2) including interaction terms between social connectedness and health literacy.

Statistical significance: *p < 0.05; **p < 0.01, ***p < 0.001.

Bold values indicate statistically significant *p*-values for the exposure variables.

**TABLE 4:** Partial associations between health literacy and the number of unhealthy lifestyle behaviours by category of social connectedness, adults aged 58+, Survey of Health, Ageing, and Retirement in Europe, 2019/2020, *n* = 1,455.

	Number of unhealthy lifestyle behaviours		Number of unhealthy lifestyle behaviours	
	Model 1		Model 2	
	No social isolation	Social isolation	No social isolation	Social isolation
**Covariates**				
Sex	−0.05 (0.05)	0.14 (0.11)	−0.06 (0.05)	0.14 (0.11)
Women (vs. men)				
Age groups				
65–74 years (vs. 58–64)	−0.09 (0.07)	0.02 (0.15)	−0.09 (0.07)	0.03 (0.15)
75+ years (vs. 58–64)	0.03 (0.07)	0.03 (0.14)	0.03 (0.08)	0.05 (0.14)
Linguistic region				
French-speaking (vs. German-speaking)	−0.00 (0.06)	0.01 (0.14)	−0.00 (0.06)	0.00 (0.14)
Italian-speaking (vs. German-speaking)	−0.13 (0.16)	0.03 (0.17)	−0.14 (0.16)	0.04 (0.17)
Education levels				
Secondary (vs. low)	0.04 (0.07)	0.01 (0.14)	0.04 (0.07)	0.00 (0.14)
Tertiary (vs. low)	−0.00 (0.09)	−0.04 (0.18)	−0.01 (0.09)	−0.04 (0.18)
Make ends meet				
Fairly easily (vs. easily)	0.13* (0.06)	0.10 (0.13)	0.14* (0.06)	0.11 (0.13)
With difficulty (vs. easily)	0.17* (0.08)	0.32* (0.15)	0.17* (0.08)	0.32* (0.15)
Living environment				
Rural (vs. urban)	−0.02 (0.05)	−0.11 (0.11)	−0.02 (0.05)	−0.12 (0.11)
Self-rated health				
Good (vs. poor/fair)	−0.20* (0.08)	−0.37* (0.16)	−0.21** (0.09)	−0.39* (0.15)
Very good/excellent (vs. poor/fair)	−0.39*** (0.09)	−0.47** (0.18)	−0.41*** (0.09)	−0.48** (0.17)
Limitations in activities of daily living				
One or more (vs. no limitations)	0.22* (0.10)	0.23 (0.15)	0.22* (0.10)	0.27 (0.14)
Prevalent major chronic diseases				
Yes (vs. no)	0.01 (0.06)	0.19 (0.13)	0.01 (0.06)	0.20 (0.13)
**Exposure variables**				
Health literacy total score (0–16)	−0.02 (0.01)	−0.03* (0.01)		
Health literacy levels				
Problematic (vs. sufficient health literacy)			0.02 (0.06)	0.13 (0.14)
Inadequate (vs. sufficient health literacy)			0.16 (0.10)	0.34* (0.15)

The table shows average partial effects and standard errors in parentheses from separate Poisson regression models for health literacy total score (Model 1) and health literacy levels (Model 2).

Statistical significance: *p < 0.05; ** *p* < 0.01, *** *p* < 0.001.

Bold values indicate statistically significant *p*-values for the exposure variables.

## Discussion

Using data from 1,455 adults aged 58 and older in the Swiss general population, we assessed the association between health literacy levels and the number of prevalent unhealthy lifestyle behaviours. We additionally explored the moderating role of social connectedness in this relationship. Independently of key socio-demographic and health characteristics, we found that respondents with problematic and inadequate health literacy levels were significantly more likely to report a higher number of unhealthy lifestyle behaviours than their counterparts with adequate health literacy. Also, we found that this relationship was somewhat stronger among respondents who were considered socially isolated, suggesting that greater social connectedness may buffer the negative impact of inadequate health literacy on unhealthy lifestyle behaviours in the target population.

These findings, which bring new evidence on the relationship between health literacy and lifestyle behaviours among older adults, partly align with existing literature. In a nationally representative sample of 707 US older adults, Fernandez et al. found that respondents with adequate self-reported health literacy were significantly more likely to engage in moderate physical activity than participants with inadequate self-reported health literacy [22]. However, the findings regarding engagement in vigorous physical activity and current smoking did not reach statistical significance [22]. In their cross-sectional study including 354 Iranian older adults, Reisi et al. found a significant relationship between higher objective health literacy levels and more frequent physical activity and higher fruit and vegetable consumption [20]. However, the authors did not conduct any multivariable analyses to account for potential confounders in these relationships. Conversely, in another cross-sectional study among 2,923 US adults aged 65+, Wolf et al. found an absence of association between inadequate objective health literacy and health risk behaviours, including self-reported cigarette smoking, alcohol consumption, and physical activity, after controlling for relevant covariates [19]. The heterogeneity in the measurement of health literacy across studies may partly explain the lack of consistency in their findings. As evidenced by [22], results on the association between lifestyle factors and health literacy may slightly differ when using subjective or objective measures of health literacy. The authors reported a significant positive relationship between self-reported health literacy and engagement in physical activity. In contrast, a significant inverse relationship was found between objective health literacy and the current use of tobacco. So far, the existing literature suggests that subjective and objective health literacy may assess related but different constructs. As objective measures of health literacy can be seen as a direct measure of an individual’s literacy capability in the context of health and their ability to accomplish certain reading and problem-solving tasks [39], these performance-based measures may be context-specific and not necessarily designed for more general studies of lifestyle behaviours. Self-rated health literacy was shown to be more related to self-efficacy [40], i.e., one’s belief in one’s ability to succeed in specific situations or accomplish a task, which may influence how people successfully deal with health information [41] and ultimately impact their health behaviours. Also, unlike all the studies mentioned above, we used the HLS-EU-Q16 questionnaire, a validated and widely recognised scale designed to measure different dimensions and provide a more holistic picture of individuals’ subjective health literacy [33, 42].

The comparability of the results of our study with those in the literature is also limited by the fact that previous works focused on individual lifestyles, although unhealthy lifestyle factors tend to co-occur [43] and generally have synergistic interactions leading to the development of chronic conditions and increased risk of mortality [44]. A recent systematic literature review including 25 studies highlighted that individuals who reported being engaged in physical activity combined with meeting other health behaviour goals, i.e., not smoking, eating healthy, and limited sedentary behaviour and alcohol consumption, had at least a 50% reduction in the risk of having cardiovascular diseases, of dying from cardiovascular diseases or dying from any cause, compared to individuals who were classified as physically inactive and did not meet other positive lifestyle goals [45]. The findings of the present study of a significant association between limited health literacy and a higher number of prevalent unhealthy lifestyle behaviours support that improving health literacy could constitute a breakthrough in promoting positive changes in health behaviour to ultimately mitigate the morbidity and mortality of chronic conditions in later life.

Our work further contributes to the literature by assessing the moderating role of social connectedness in the relationship between health literacy and lifestyle behaviours. To date, only one study by [21] has examined this question, reporting that the associations of health literacy with physical activity, fruit consumption, vegetable consumption, and alcohol consumption were not significantly moderated by any of the studied social factors, i.e., loneliness, social support, living situation, engagement in social activities and the number of social contacts. The only significant moderator of the association between health literacy and smoking behaviour was the number of social contacts. Beyond the differences in our study outcomes, it may be possible that our findings diverge from those of Geboers et al. in part due to the distinct operationalisation of the variable related to social connectedness. Considering the functional characteristics of social networks, such as provided or perceived available support, as important aspects of social connectedness may potentially be ambivalent as individuals may not need support at a specific time [46]. In addition, focusing on the influence of network size may overlook the importance of the quality of the relationships maintained. As the social connectedness scale used in the present study provides both quantitative and qualitative aspects of the social network in a single measure, it provides insights into meaningful relationships of older adults, which could help to identify more accurately possible isolated older adults in the population [35]. Our findings, which indicate a stronger positive association between inadequate health literacy and the number of prevalent unhealthy lifestyle behaviours among socially isolated respondents, are consistent with the theory that health outcomes, health-related behaviours, and health literacy should be placed in the context of the personal and socio-physical environments of individuals [47]. Building on the theory of social capital introduced by Bourdieu in the 80s [48], recent conceptual frameworks have notably focused on the importance of “social reserve,” referring to the accumulated social resources that individuals possess, which can act as a buffer during times of adversity or stress, promoting better health outcomes in older age [49, 50]. Studies focusing specifically on the interplay between social context and health literacy have suggested that health literacy functions more as a social practice than simply an individual competence [26, 27]. This perspective highlights the influential roles of other individuals, families, and communities in one’s health information acquisition, comprehension, and decision-making [51]. Notably, Edwards et al. introduced the “distributed health literacy” model that argues that while individual health literacy may vary within a group, individuals can overcome personal deficits in health literacy skills by combining their efforts [24]. In this way, distributed health literacy could be considered a resource that may buffer the adverse impacts of low health literacy. Interventions considering the social context of health literacy and ensuring that people have both informed networks and the skills to draw on them could help reduce health disparities, especially among older adults who often have caregivers [51, 52]. A recent systematic literature review suggested that existing interventional studies aimed at improving health literacy skills significantly improved several behavioural outcomes such as smoking prevention behaviours, nutrition-related behaviours, and physical activity behaviours [53]. However, given the few existing studies, the authors stressed the need to continue developing new health literacy interventions that make better use of behaviour change theory to more effectively improve participants’ health literacy, which, in turn, may help behaviour change interventions to be more effective [53]. Additionally, the development and implementation of new health-promoting lifestyle interventions conducted among older adults remain important to, for instance, strengthen the evidence on the benefits of joining sporting clubs or community groups offering different activity options on physical activity levels [54].

Although our findings provide new evidence on the importance of good health literacy and social reserve in making healthy lifestyle choices in the older adult population, some limitations need to be acknowledged. The present study used data from a population-based survey with a high response rate. However, we cannot entirely rule out the existence of a potential residual selection bias. Indeed, some vulnerable population subgroups at high risk of low health literacy, such as the oldest-old adults or individuals with severe health may be underrepresented among the survey respondents. Therefore, the potential residual selection bias may have resulted in an overestimation of the health literacy skills of the sample, making the estimates of the observed associations conservative. Also, given the self-reported nature of the assessment of the different lifestyle factors, we cannot rule out the possibility of social desirability bias. Indeed, respondents could tend to underreport socially undesirable behaviours, leading to a potential underestimation of the prevalence of unhealthy lifestyle behaviours in the study population. Additionally, the observed difference in the number of unhealthy behaviours between respondents with inadequate versus sufficient health literacy may seem marginal. Nonetheless, when viewed in the context of a broader population, such a difference may potentially be associated with a higher prevalence of overall unhealthy behaviours and related morbidity and mortality. Lastly, causality cannot be inferred because of the cross-sectional and observational nature of our study.

### Conclusion

In conclusion, the present study’s findings suggest that greater social connectedness may buffer the negative impact of low health literacy on the number of unhealthy lifestyle behaviours, highlighting the importance of good health literacy for healthy lifestyles, especially in individuals with low social reserve. Given the potential spillover effects that health literacy may have on others through its potential transmission in social networks, improving health literacy skills could result in older adults being better able to seek information, have the confidence to apply it and ultimately manage their lifestyles. Further, implementing health-promoting lifestyle interventions including a family or community component to strengthen social connections and mitigate isolation as well as tailoring communication and health education to different health literacy levels would help tackle the significant burden of low health literacy and unhealthy lifestyle behaviours in older age.

## Data Availability

The datasets generated and/or analysed during the current study are available to the scientific community upon submitting a data requestion application to the SHARE study (https://share-eric.eu/data/become-a-user). Additional materials can be received upon request on: maud.wieczorek@unil.ch.
